# Is data cleaning and the testing of assumptions relevant in the 21st century?

**DOI:** 10.3389/fpsyg.2013.00370

**Published:** 2013-06-25

**Authors:** Jason W. Osborne

**Affiliations:** Educational and Counseling Psychology, University of LouisvilleLouisville, Kentucky, USA

You must understand fully what your assumptions say and what they imply. You must not claim that the “usual assumptions” are acceptable due to the robustness of your technique unless you really understand the implications and limits of this assertion in the context of your application. And you must absolutely never use any statistical method without realizing that you are implicitly making assumptions, and that the validity of your results can never be greater than that of the most questionable of these (Vardeman and Morris, 2003, p. 26).

Modern quantitative studies use sophisticated statistical analyses that rely upon numerous important assumptions to ensure the validity of the results and protection from mis-estimation of outcomes. Yet casual inspection of respected journals in various fields shows a marked absence of discussion of the mundane, basic staples of quantitative methodology such as data cleaning or testing of assumptions, leaving us in the troubling position of being surrounded by intriguing quantitative findings but not able to assess the quality or reliability of the knowledge base of our field.

Few of us become scientists in order to do harm to the literature. Indeed most of us seek to help people, improve the world in some way, to make a difference. However, all the effort in the world will not accomplish these goals in the absence of valid, reliable, generalizable results—which can only be had with clean (non-faulty) data and assumptions of analyses met.

## Where does this idea of data cleaning and testing assumptions come from?

Researchers have discussed the importance of assumptions from the introduction of our early modern statistical tests (e.g., Pearson, 1901; Student, 1908; Pearson, 1931). Even the most recently-developed statistical tests are developed in a context of certain important assumptions about the data.

Mathematicians and statisticians developing the tests we take for granted today had to make certain explicit assumptions about the data in order to formulate the operations that occur “under the hood” when we perform statistical analyses. A common example is that the data (or errors) are normally distributed, or that all groups (errors) have roughly equal variance. Without these assumptions the formulae and conclusions are not valid.

Early in the 20th century these assumptions were the focus of vigorous debate and discussion. For example, since data rarely are perfectly normally distributed, how much of a deviation from normality is acceptable? Similarly, it is rare that two groups would have exactly identical variances, how close to equal is good enough to maintain the goodness of the results?

By the middle of the 20th century, researchers had assembled some evidence that some *minimal* violations of some assumptions had minimal effects on error rates under certain circumstances—in other words, if your variances are not exactly identical across all groups, but are relatively close, it is probably acceptable to interpret the results of that test despite this technical violation of assumptions. Box (1953) is credited with coining the term “robust” (Boneau, 1960) which usually indicates that violation of an assumption does not substantially influence the Type I error rate of the test^1^. Thus, many authors published studies showing that analyses such as simple one-factor ANOVA analyses are “robust” to non-normality of the populations (Pearson, 1931) and to variance inequality (Box, 1953) when group sizes are equal. This means that they concluded that modest (practical) violations of these assumptions would not increase the probability of Type I errors [although even Pearson (1931) notes that strong non-normality can bias results toward increased Type II errors].

These fundamental, important debates focused on minor (but practically insignificant) deviations from absolute normality or exactly equal variance, (i.e., if a skew of 0.01 or 0.05 would make results unreliable). Despite being relatively narrow in scope (e.g., primarily concerned with Type I error rates in the context of exactly equal sample sizes and relatively simple one-factor ANOVA analyses) these early studies appear to have given social scientists the impression that these basic assumptions are unimportant. These early studies do not mean, however, that *all* analyses are robust to *dramatic* violations of these assumptions, or attest to robustness without meeting the other conditions (e.g., exactly equal cell sizes).

These findings do not necessarily generalize to broad violations of any assumption under any condition, and leave open questions regarding Type II error rates and mis-estimation of effect sizes and confidence intervals. Unfortunately, the latter point seems to have been lost on many modern researchers. Recall that these early researchers on “robustness” were often applied statisticians working in places such as chemical and agricultural companies as well as research labs such as Bell Telephone Labs, not in the social sciences where data may be more likely to be messy. Thus, these authors are viewing “modest deviations” as exactly that- minor deviations from mathematical models of perfect normality and perfect equality of variance that are practically unimportant. Social scientists rarely see data that are as clean as that discussed in these robustness studies.

Further, important caveats came with conclusions around “robustness”—such as adequate sample sizes, equal group sizes, and relatively simple analyses such as one-factor ANOVA.

This mythology of robustness, however, appears to have taken root in the social sciences and may have been accepted as broad fact rather than narrowly, as intended. Through the latter half of the 20th century this term came to be used more often as researchers published narrowly-focused studies that appeared to reinforce the mythology of robustness, perhaps inadvertently indicating that robustness was the rule rather than the exception.

In one example of this type of research, studies reported that simple statistical procedures such as the Pearson Product-Moment Correlation and the One-Way ANOVA (e.g., Feir-Walsh and Toothaker, 1974; Havlicek and Peterson, 1977) were robust to even “substantial violations” of assumptions. It is perhaps not surprising that “robustness” appears to have become unquestioned canon among quantitative social scientists, despite the caveats to these latter assertions, and the important point that these assertions of robustness usually relates only to Type I error rates, yet other aspects of analyses (such as Type II error rates or the accuracy of the estimates of effects) might still be strongly influenced by violation of assumptions.

However, the finding that simple correlations might be robust to certain violations is not to say that similar but more complex procedures (e.g., multiple regression, path analysis, or structural equation modeling) are equally robust to these same violations. Similarly, should one-way ANOVA be robust to violations of assumptions^2^, it is not clear that similar but more complex procedures (e.g., factorial ANOVA or ANCOVA) would be equally robust to these violations. Yet recent surveys of quantitative research in many sciences affirms that a relatively low percentage of authors in recent years report basic information such as having checked for extreme scores, normality of the data, or having tested assumptions of the statistical procedures being used (Keselman et al., 1998; Osborne, 2008; Osborne et al., 2012). It seems, then, that this “mythology of robustness” has led a substantial percentage of social science researchers to believe it unnecessary to check the goodness of their data and the assumptions that their tests are based on (or report having done so).

Recent surveys of top research journals in the social sciences^3^ confirm that authors (and reviewers and editors) are disconcertingly casual about data cleaning and reporting of tests of assumptions. One prominent review of education and psychology research by Keselman et al. (1998) provided a thorough review of empirical social science during the 1990s. The authors reviewed studies from 17 prominent journals spanning different areas of education and psychology, focusing on empirical articles with ANOVA-type designs.

In looking at 61 studies utilizing univariate ANOVA between-subjects designs, the authors found that only 11.48% of authors reported anything related to assessing normality, almost uniformly assessing normality through descriptive rather than inferential methods. Further, only 8.20% reported assessing homogeneity of variance, and only 4.92% assessed both distributional assumptions and homogeneity of variance. While some earlier studies asserted ANOVA to be robust to violations of these assumptions (Feir-Walsh and Toothaker, 1974), more recent work contradicts this long-held belief, particularly where designs extend beyond simple One-Way ANOVA and where cell sizes are unbalanced (which seems fairly common in modern ANOVA analyses within the social sciences) (Wilcox, 1987; Lix et al., 1996).

In examining articles reporting multivariate analyses, Keselman et al. (1998) describe a more dire situation. None of the 79 studies utilizing multivariate ANOVA procedures reported examining relevant assumptions of variance homogeneity, and in only 6.33% of the articles was there any evidence of examining of distributional assumptions (such as normality).

Similarly, in their examination of 226 articles that utilized some type of repeated-measures analysis, only 15.50% made reference to some aspect of assumptions, but none appeared to report assessing sphericity, an important assumption in these designs that can lead to substantial inflation of error rates and mis-estimation of effects, when violated (Maxwell and Delaney, 1990, p. 474).

Finally, their assessment of articles utilizing covariance designs (*N* = 45) was equally disappointing—75.56% of the studies reviewed made no mention of any assumptions or sample distributions, and most (82.22%) failed to report any information about the assumption of homogeneity of regression slope, an assumption critical to the validity of ANCOVA designs.

Another survey of articles published in 1998 and 1999 volumes of well-respected Educational Psychology journals (Osborne, 2008) showed that indicators of high quality data cleaning in published articles were sorely lacking. Specifically, authors in these top educational psychology journals almost never reported testing any assumptions of the analyses used (only 8.30% reported having tested any assumption), only 26.0% reported reliability of data being analyzed, and none reported any significant data cleaning (e.g., examination of data for outliers, normality, analysis of missing data, random responding, etc.).

Finally, a recent survey of recent articles published in prominent APA journals 2009 volumes (Osborne et al., 2012) found improved, but uninspiring results (see Figure 1.1). For example, the percentage of authors reporting anything resembling minimal data cleaning ranged from 22 to 38% across journals. This represents a marked improvement from previous surveys, but still leaves a majority of authors failing to report any type of data cleaning or testing of assumptions, a troubling state of affairs. Similarly, between 10 and 32% reported checking for distributional assumptions, and 32–45% reported dealing with missing data in some way (although usually through methods considered sub-optimal). Clearly, even in the 21st century, the majority of authors in highly-respected scholarly journals fail to report information about these basic issues of quantitative methods.

When I wrote a whole book on data cleaning (Osborne, 2012), my goal was to debunk this mythology of robustness and *laissez-faire* that seems to have seeped into the zeitgeist of quantitative methods. The challenge handed to authors in this book was to go beyond the basics of data cleaning and testing assumptions—to show that assumptions and quality data are still relevant and important in the 21st century. They went above and beyond this challenge in many interesting—and unexpected ways. I hope that this is the beginning—or a continuation—of an important discussion that strikes at the very heart of our quantitative disciplines; namely, whether we can trust any of the results we read in journals, and whether we can apply (or generalize) those results beyond the limited scope of the original sample.

## Footnotes

^1^Note that Type II error rates and mis-estimation of parameters is much less rarely discussed and investigated.

^2^To be clear, it is debatable as to whether these relatively simple procedures are as robust as previously asserted.

^3^Other reviewers in other sciences tend to find similar results, unfortunately.
